# Association Between Sense of Coherence and Frailty: A Cross-Sectional Study in China

**DOI:** 10.3389/fpsyt.2022.844439

**Published:** 2022-04-05

**Authors:** Hao Chen, Hua Fu, Bo Ye, Yi Wang, Huihui Yan, Yingwei Chen, Jixiang Xu, Xin Nie, Junling Gao

**Affiliations:** ^1^Department of Preventive Medicine and Health Education, School of Public Health, Fudan University, Shanghai, China; ^2^Department of Geriatric Medicine, Huadong Hospital Affiliated to Fudan University, Shanghai, China

**Keywords:** frailty, sense of coherence, healthy ageing, older adults, community

## Abstract

**Purpose:**

Frailty is an emerging global public health burden. Most existing studies have focused on risk factors for frailty, focusing less on protective factors against frailty. This study aims to examine the association between the sense of coherence (SOC), the most common construct of salutogenesis and frailty status among community-dwelling old adults.

**Method:**

A cross-sectional study was conducted among 7,970 old adults aged ≥65 years in three cities in China from June 2019 to October 2020. Frailty was operationalised as the sum of self-reported fatigue, resistance, ambulation, illness, and loss of weight (FRAIL scale). The χ^2^ test was used to analyse the distribution difference of frailty in demographic, behavioural, and SOC levels. Confounder-adjusted multinomial logistic regression was used to examine the association between SOC and frailty.

**Results:**

The prevalence of pre-frailty and frailty was 43.1 and 8.0%, respectively. The results of the confounder-adjusted regression showed that older adults with moderate-level SOC (odds ratio, OR: 0.61, 95% CI: 0.54–0.69) and strong-level SOC (OR: 0.55, 0.48–0.64) had lower odds of being pre-frail compared to those with weak SOC. It also showed that older adults with moderate-level SOC (OR: 0.32, 95% CI: 0.27–0.40) and strong-level SOC (OR: 0.22, 95% CI: 0.16–0.29) had lower odds of being frail compared to those with weak SOC.

**Conclusion:**

SOC may be a protective factor against frailty. Improving SOC may be a strategy to prevent frailty among Chinese community-dwelling older adults.

## Introduction

Frailty is a geriatric syndrome characterised by non-specific vulnerability to adverse events (e.g., mortality, institutionalisation, falls, hospitalisation), which is attributed to the deregulation of multiple and complex physiological system factors associated with advancing age (1–3). The biological basis of frailty is multifactorial, involving multiple etiologic dysregulations across many physiological systems, cumulative cellular damage, inflammation, malnutrition, and sarcopenia (4). The cycle of frailty described by Fried et al. also considers the effects of behaviours and environmental determinants on deteriorated frailty states, including inadequate nutrition intake, physical inactivity, and stressful life events (5).

Research has found that frailty heterogeneity exists among older adults of the same age group with the same risk factors (6, 7). To determine whether health heterogeneity was the initial purpose of *salutogenesis*, a theory proposed by Aron Antonovsky in the late 1970s, raises the question of which salutary factors actively maintain or promote health and which risk factors cause disease (8, 9). *Salutogenisis* argued that the human system is inherently flawed, subject to unavoidable entropic processes and unavoidable final death, which follows a continuum health model of ease/dis-ease movement rather than dichotomous classification (health and illness) (6). Sense of coherence (SOC), the most important construct of *salutogenesis*, refers to an orientation toward life that characterises the extent to which an individual appraises internal and external environments as comprehensible, manageable, and meaningful (10). This would influence the dynamically continuous movement between ease (robust) and dis-ease (frail). The three components of SOC are comprehensibility, manageability, and meaningfulness, which reflect their respective (1) perception of internal and external stimuli as structured, predictable and explicable; (2) conviction that one has the available resources to meet the demands of these stimuli; and (3) belief that all of these demands have a reason and are worth challenging (8). SOC is a critical salutary health factor. Studies found that strong SOC has a protective effect against negative health outcomes in terms of depression, (11) low quality of life, (12) disability, (13) and mortality (14, 15) as well as toward an ease status. In addition, SOC is malleable and can be enhanced by appropriate interventions in the older adult population (16). Although some studies found that older adults with strong SOC had less physical functional decline (17) and more cognitive functional reserve, (18) few studies have examined the relationship between SOC and frailty. Therefore, the present study aimed to examine whether SOC was associated with frailty status among community dwelling older adults aged ≥65 years.

## Materials and Methods

### Participants and Study Design

This cross-sectional study was conducted in three cities in China: Shanghai (Southern China), Tianjin (Northern China), and Ordos (Northern China) from June 2019 to October 2020. A total of 8,590 community-dwelling older adults were randomly recruited from 31 districts using a multistage stratified sampling method, among which 16 communities were in Shanghai, 6 in Tianjin, and 9 in Ordos. The minimum sample size of each selected community was required to have no less than 200 participants. The general practitioners from each selected community visited participants in their homes or invited them to community healthcare centres by using uniform questionnaires after obtaining informed consent. Inclusion criteria were as follows: (1) residing in the community for more than 6 months and (2) aged 65 or older. Exclusion criteria were as follows: (1) severe psychological disorders and (2) an inability to answer questions. We ultimately included 7,970 (92.8%) participants in the present study after excluding incomplete data. The Ethics Committee for Medical Research at the School of Public Health, Fudan University, approved this study (IRB00002408 and FWA00002399).

### Measurements

#### Frailty

The Chinese version of the FRAIL scale was used to measure Frailty. The scale consists of five “yes/no” items assessing five different functional ability domains (Fatigue, Resistance, Ambulation, Illness, and Loss of weight), (19) which is a validated and widely used screening tool to identify frail or prefrail individuals in over 15 countries, including China (20). Frailty scores range from zero to five (i.e., one point for each component; 0 = *best* to 5 = *worst*) and represent robust (0), pre-frail (1–2), and frail (3–5).

#### Sense of Coherence

The Chinese version of the SOC scale (C-SOC-13) with acceptable reliability and validity consists of three dimensions: comprehensibility (five items), manageability (four items), and meaningfulness (four items), and was used to measure the level of SOC (21). Each item is scored on a seven-point Likert scale, ranging from 1 (*very often*) to 7 (*never or very seldom*). The total SOC score is obtained by summing the corresponding item scores after revising the five negatively worded items, with a higher SOC score indicating a preferable sense of coherence level (22). In the present study, Cronbach’s alpha coefficient for the internal consistency of the C-SOC-13 was 0.88, and the SOC score was categorized into tertiles for weak, moderate, and strong levels (14).

#### Covariates

Based on the literature, (23–25) covariates in this study included age (5-year categories), sex (male and female), marital status (married or cohabiting vs. other), educational attainment (illiteracy, primary, junior high school, and above), location (Southern and Northern China), and health-related behaviours including smoking, drinking, physical activity, vegetable intake, and fruit intake.

Smoking status was assessed using two questions: “Q1. Have you ever smoked over 100 cigarettes? (yes/no); Q2. Have you smoked in the past 30 days? (yes/no).” Participants who answered “yes” to both questions were classified as smokers; otherwise, they were classified as non-smokers.

Drinking status was derived from frequency responses (never/once per month or less/2–4 times per month/2–3 times per week/over four times per week) to the question “How often do you drink alcohol?” Participants who answered “never” were classified as non-drinkers; otherwise, they were classified as drinkers.

Physical activity was assessed using two questions: “Q1. How many times did you participate in moderate-intensity physical activity (heart rate and breathing rate increase and slight perspiration) per week? (None, 1–2 times, 3–4 times, 5–6 times, seven times or more); Q2. For how long did you participate each time? (less than 20 min, 20–30 min, 30–40 min, 40–50 min, or more than 50 min)” (26). In accordance with the current recommendations for the practising of physical activity, this study classified participants with at least 150 min of moderate physical activity per week as physically active, while other participants were physically inactive (27).

Vegetable intake was derived from weight responses (0–200 g, 200–300 g, 300–400 g, 400–500 g, and over 500 g) to the question “On average, how much fruit do you eat per day?” (28). Fruit intake was also derived from weight responses (0–100, 100–200, 200–350, 350–500, and over 500 g) to the question “On average, how much fruit do you eat per day?” (28). In accordance with the current recommendations for the Chinese Dietary Guidelines, the present study defined at least 300 g of vegetable intake and 200 g of fruit intake as adequate intake (29).

### Statistical Analysis

Firstly, we used descriptive analysis to show the characteristics frailty states and SOC of participants, and then ANOVA test and multiple-comparisons (Bonferroni method) were used to examine the difference distribution of SOC according to frailty states (robust, pre-frail, and frail). Secondly, χ^2^ tests were used to examine the distribution of frailty states according to demographic characteristics, health-related behaviours, and ranked SOC (weak, moderate, and strong). Furthermore, multinomial logistic regression models were used to examine the associations between SOC and pre-frailty (Model a1) and frailty (Model a2) after adjusting for age, sex, marital status, educational attainment, and location. Then, health-related behaviours were added to Models a1 and a2 in order to examine the associations between SOC and pre-frailty (Model b1) and frailty (Model b2). The estimates of SOC and health-related behaviours for frailty were summarized using odds ratios (Ors) and their 95% confidence intervals (Cis). Statistical analyses were performed using the R software (version 4.1.1) (25).

## Results

### Descriptive Results of Demographic Characteristics, Frailty, and Sense of Coherence

As shown in Table 1, the average age of 7,970 participants was 72.33 years (SD: 6.00, Range: 65–101); 52.7% of them were female, and nearly half of the participants were illiterate (43.5%). The majority of the participants (81.6%) were married or co-inhabited. The prevalence of smoking and drinking was 24.0 and 12.8%, respectively. Over half of the participants (60.1%) reported that they were physically inactive. Inadequate vegetable and fruit intake was reported by 47.2 and 74.9% of participants, respectively.

**TABLE 1:** The frailty stage distribution in demographic characteristic, SOC, and behaviours (*n* = 7,970).

Variable	Total [n(%)]	Frailty stage [n(%)]			*P*-value
		Robust	Pre-frail	Frail	
**Age (years)**					**<0.001**
65–69	3159(39.6)	1739(55.0)	1263(40.0)	157(5.0)	
70–74	2362(29.6)	1214(51.4)	987(41.8)	161(6.8)	
75–79	1401(17.6)	597(42.6)	648(46.3)	156(11.1)	
>80	1048(13.2)	350(33.4)	535(51.0)	163(15.6)	
**Sex**					**<0.001**
Male	3766(47.3)	1983(52.7)	1542(40.9)	241(6.4)	
Female	4204(52.7)	1917(45.6)	1891(45.0)	396(9.4)	
**Education level**					**<0.001**
Illiteracy	3470(43.5)	1457(42.0)	1673(48.2)	340(9.8)	
Primary school	2515(31.6)	1381(54.9)	978(38.9)	156(6.2)	
≥Junior-senior high school	1985(24.9)	1062(53.5)	782(39.4)	141(7.1)	
**Marital status**					**< 0.001**
Married	6502(81.6)	3317(51.0)	2738(42.1)	447(6.9)	
Not married	1468(18.4)	583(39.7)	695(47.3)	190(12.9)	
**SOC**					**<0.001**
Weak	1864(23.4)	645(34.6)	932(50.0)	287(15.4)	
Moderate	4280(53.7)	2200(51.4)	1804(42.1)	276(6.4)	
Strong	1826(22.9)	1055(57.8)	697(38.2)	74(4.1)	
**Smoke**					**<0.001**
Non-smoker	6061(76.0)	2851(47.0)	2686(44.3)	524(8.6)	
Smoker	1909(24.0)	1049(55.0)	747(39.1)	113(5.9)	
**Drink**					**<0.001**
Non-drinker	6946(87.2)	3280(47.2)	3072(44.2)	592(8.7)	
Drinker	1024(12.8)	620(60.5)	361(35.3)	43(4.2)	
**Physical activity**					**<0.001**
Physical inactivity	4792(60.1)	2016(42.1)	2261(47.2)	515(10.7)	
Physically active	3178(39.9)	1884(59.3)	1172(36.9)	122(3.8)	
**Vegetable intake**					**<0.001**
Inadequate	3764 (47.2)	1537(40.8)	1796(47.7)	431(11.5)	
Adequate	4204 (52.8)	2363(56.2)	1637(38.9)	206(4.9)	
**Fruit intake**					**<0.001**
Inadequate	5967(74.9)	2664(44.6)	2761(46.3)	542(9.1)	
Adequate	2003(25.1)	1236(61.7)	672(33.5)	95(4.7)	

*Bold values are statistical difference were significant (p < 0.05).*

**FIGURE 1:**
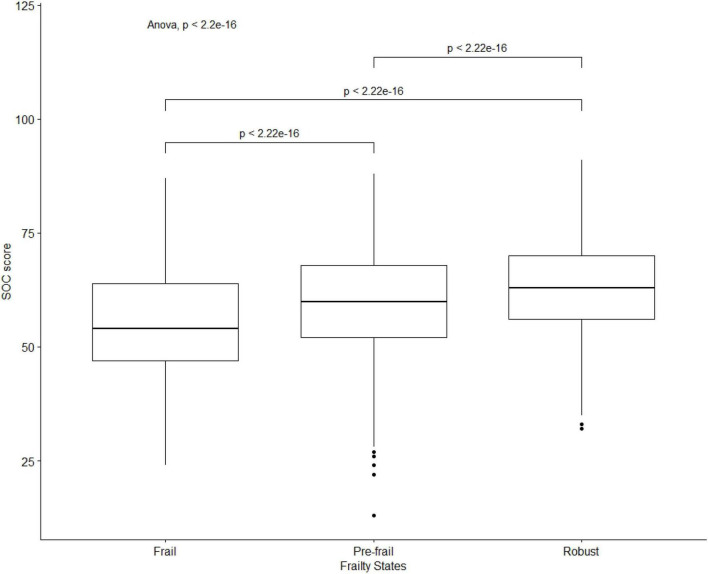
A boxplot of the distribution of SOC score among frail, pre-frail, and robust participants.

As for the frailty states, 48.9% of participants were robust, 43.1% were pre-frail, and 8.0% were frail. The mean score of SOC was 60.80 (SD: 11.00, Range: 13–91), and its distribution among different frailty states examined by using ANOVA test and multiple-comparisons is shown in Figure 1. The mean score of SOC among frail participants (mean: 54.99; SD: 11.52) was lower than pre-frail participants (mean: 59.38; SD: 10.75) and robust participants (mean: 63.00; SD: 10.56), *p* < 0.001.

### Univariate Analysis for Frailty Distribution

As shown in Table 1, the univariate analysis results indicated that the distribution of frailty showed statistical differences in age, sex, education attainment, marital status, SOC, smoking, drinking, physical activity, vegetable intake, and fruit intake (all *P* < 0.001). The prevalence of frailty significantly decreased with decreasing age (5-year categories) and with increased SOC levels (strong to moderate to weak). The prevalence of frailty among those who were unmarried (12.9%) and females (9.4%) was higher than among married (6.9%) and male (6.4%) older adults. Compared with illiteracy elders (9.8%), the frailty prevalence was lower among elders who received primary (6.2%) and junior-senior high school and above (7.1%). Frailty prevalence was lower among smokers (5.9%) and drinkers (4.2%) than among non-smokers (8.6%) and non-drinkers (8.7%). The prevalence of frailty was higher among the physically inactive (10.7%), those with inadequate vegetable intake (11.5%), and those with inadequate fruit intake (9.1%) than among those who were physically active (3.8%), had adequate vegetable intake (4.9%), and those with adequate fruit intake (4.7%). The prevalence of pre-frailty among participants with weak SOC was 50.0%, while that among participants with moderate and strong SOC was 42.1 and 38.2%, respectively. The prevalence of frailty among participants with weak SOC was 15.4%, while that among moderate and strong SOC participants was 6.4 and 4.1%, respectively.

### Multivariate Analysis for Associations Between Sense of Coherence and Frailty

The results of the confounder-adjusted multinomial logistic regression models for associations of SOC with the odds of being pre-frail and frail are shown in Table 2. In the adjusted Model 2, older adults with moderate SOC (odds ratio, OR: 0.61, 95% CI: 0.54–0.69) and strong SOC (OR: 0.55, 95% CI: 0.48–0.64) levels had lower odds of being pre-frail compared to those with weak SOC, respectively, *P* < 0.001; older adults who had moderate SOC (OR: 0.32, 95% CI: 0.27–0.40) and strong SOC (OR: 0.22, 95% CI: 0.16–0.29) levels had lower odds of being frail compared to those with weak SOC, respectively, *P* < 0.001.

**TABLE 2:** The associations between SOC and frailty by using multinomial logistic regressions.

Variable	Model 1 (Robust as ref.)		Model 2 (Robust as ref.)	
	Pre-frail	Frail	Pre-frail	Frail
**SOC**				
Weak	1 (ref.)	1 (ref.)	1 (ref.)	1 (ref.)
Moderate	0.59(0.53–0.67)***	0.29(0.24–0.36)***	0.61(0.54–0.69)***	0.32(0.27–0.40)***
Strong	0.52(0.45–0.60)***	0.18(0.14–0.24)***	0.55(0.48–0.64)***	0.22(0.16–0.29)***
**Smoke**				
Non-smoker			1 (ref.)	1 (ref.)
Smoker			0.88(0.78–1.01)	0.84(0.63–1.07)
**Drink**				
Non-drinker			1 (ref.)	1 (ref.)
Drinker			0.81(0.69–0.95)**	0.69(0.48–0.99)*
**Physical activity**				
Physical inactivity			1 (ref.)	1 (ref.)
Physically active			0.63(0.57–0.70)***	0.35(0.28–0.44)***
**Vegetable intake**				
Inadequate			1 (ref.)	1 (ref.)
Adequate			0.77(0.70–0.86)***	0.48(0.39–0.58)***
**Fruit intake**				
Inadequate			1 (ref.)	1 (ref.)
Adequate			0.63(0.56–0.71)***	0.62(0.48–0.80)***

ref., reference; *, P < 0.05; **, P < 0.01; ***, P < 0.001. Model 1 include SOC and age, sex, education level, and marital status; Model 2 adds smoking, drinking, physical activity, vegetable intake, and fruit intake based on Model 1.

Furthermore, older adults who were drinkers (OR: 0.81, 95% CI: 0.69–0.95; *P* = 0.010) or physically active (OR: 0.63, 95% CI: 0.57–0.70; *P* < 0.001), had adequate vegetable intake (OR: 0.77, 95% CI: 0.70–0.86; *P* < 0.001), or adequate fruit intake (OR: 0.63, 95% CI: 0.56–0.71; *P* < 0.001) had lower odds of being pre-frail compared to those who were physically inactive or had inadequate fruit intake, respectively. Similar results were shown in the association between health-related behaviours and frailty (robust vs. frail). Older adults who were drinkers (OR: 0.69, 95% CI: 0.47–0.99; *P* = 0.041) or were physically active (OR: 0.35, 95% CI: 0.28–0.44; *P* < 0.001), had adequate vegetable intake (OR: 0.48, 95% CI: 0.39–0.58; *P* < 0.001), or had adequate fruit intake (OR: 0.62, 95% CI: 0.48–0.80; *P* < 0.001) had lower odds of being frail.

## Discussion

Preventing and even reversing frailty is crucial to achieving healthy ageing which emphasises positive processes for strengthening older adults to adapt and compensate for the negative consequences of ageing (30–32). On the one hand, *salutogenesis* focuses on searching for these positive determinants or factors that strengthen the ability to cope with intrinsic capacity decline, (32) while the main goal of healthy ageing is to maintain intrinsic capacity and delay its loss (30). On the other hand, intrinsic capacity and frailty might represent the two faces of the same coin among the elderly (31). Furthermore, some resilience factors (e.g., psychology resilience and SOC) were regarded as potential reserves of functional ability in the face of adversity (7). Therefore, it is justified to apply the theory of *salutogenesis* to frailty. The present study found a decreased prevalence of pre-frailty and frailty with advanced SOC levels. In all confounder-adjusted multivariate analysis models, it was also found that high and moderate SOC were negatively associated with the OR of frailty. From the perspective of frailty, SOC may directly affect the physiological response through an allostatic load process to stress triggered by frailty (33, 34). Besides this intermediate way to the frailty process directly, there are some explanations for why SOC may protect robust older adults from frail deterioration by reducing the risk of frailty-related psychological and physical diseases. Previous studies have reported that the protective effect of high SOC could reduce the risk of depression and anxiety, (35, 36) which are recognised as crucial indicators of frailty (37, 38). SOC was also found to be negatively associated with comorbidity, (39) which is an important component of frailty constructs (40). In addition, the SOC score tended to show a relationship with the inflammatory mediators (serum C-reactive protein and IL-6) in older adults, (41) both of which were significantly higher in pre-frailty and frail older adults than in robust older adults (42). A systematic review concluded Saultogenic-based interventions among older adults, which aimed to enhance SOC level by empowering self-management and strengthening utilisation of resource, were found to be beneficial to promotion of quality of life (43). A resistance training intervention in older adults, where resistance moving was a component of the FRAIL scale, found that participants with weak SOC before intervention may not benefit as much from training as those with strong SOC (44). Besides resistance, strong SOC was associated with decreased risks of fatigue and comorbidity (illness) which also are components of FRAIL scale in two longitudinal studies among Swedish older adults (45, 46).

The three domains of SOC (comprehensibility, manageability, and meaningfulness) may play different but reciprocal roles in frailty progression. As the vicissitudes of growing old independently strike seniors, the ageing-related process becomes unpredictable and uncontrollable for them (47). Older adults with higher comprehensibility may be more inclined to accept internal or external environmental changes as natural processes, attributing them to fate, such as poor mobility or the shrinking of social nets (48). In a salutogenic model of health, this process is a type of mechanism that promotes health status by “defining stimuli as non-stressors”(49). Seniors with strong manageability believe that they can confront stressors successfully and know how to mobilise resources to deal with risk factors for health (49). It has been reported that older adults need to engage in pursuits that are worthwhile and desirable to achieve a general view of healthy ageing to maintain a healthy status and delay deterioration (50). Furthermore, the three components are dynamically dependent, which refers to comprehensibility as a cognitive component, manageability as an instrumental/behavioural component, and meaningfulness as a motivational component (51). For example, manageability in adopting protective behaviours for frailty (e.g., physical activity and nutrition intake) can be supported by supplementing this knowledge with comprehensibility and meaningfulness, which provides older adults with the motivation to adopt frailty prevention behaviours.

There are some limitations in our study. First, the cross-sectional study design could not calculate the causal relationship between SOC and frailty. Moreover, although we controlled for demographic characteristics and behavioural covariates, we cannot exclude the possibility of residual confounding caused by unmeasured factors.

## Conclusion

This cross-sectional study elucidated a negative association between the sense of coherence and frailty. Much more research needs to be done to examine the causal relationship between the sense of coherence and frailty and how to enhance the sense of coherence among older adults.

## Data Availability Statement

The raw data supporting the conclusions of this article will be made available by the authors, without undue reservation.

## Ethics Statement

The studies involving human participants were reviewed and approved by The Research Ethics Committee of the Medical Research at the School of Public Health, Fudan University, approved the study protocol (IRB00002408 and FWA00002399). The patients/participants provided their written informed consent to participate in this study.

## Author Contributions

JG and HF designed the study and obtained the data. HC undertook the analysis supervised by JG and wrote the manuscript. JG organized the manuscript. BY helped HC in data topic selection. HC, BY, YW, HY, YC, JX, and XN performed the survey. All authors read the final manuscript and agreed with the text.

## Conflict of Interest

The authors declare that the research was conducted in the absence of any commercial or financial relationships that could be construed as a potential conflict of interest.

## Publisher’s Note

All claims expressed in this article are solely those of the authors and do not necessarily represent those of their affiliated organizations, or those of the publisher, the editors and the reviewers. Any product that may be evaluated in this article, or claim that may be made by its manufacturer, is not guaranteed or endorsed by the publisher.
